# Repurposing residential facilities for child abuse prevention: case study of the Mie Hot Spot Model for multiple-birth families in Japan

**DOI:** 10.3389/fpubh.2026.1884254

**Published:** 2026-07-03

**Authors:** Satsuki Matsumoto

**Affiliations:** Faculty of Policy Management, Yokkaichi University, Yokkaichi, Mie, Japan

**Keywords:** child abuse prevention, multiple-birth families, repurposing, residential facilities, social respite

## Abstract

**Introduction:**

Multiple-birth families (having twin, triplet, or larger births) face a disproportionately high-risk of child abuse resulting from chronic sleep deprivation and social isolation. However, community-based support often fails to address the fundamental need of such families for physiological rest. This policy note describes the Mie Hot Spot Model, an innovative intervention developed in Mie Prefecture, Japan, which repurposes maternity homes (*boshi seikatsu shien shisetsu*) into community respite hubs.

**Methods:**

By bringing underused residential capacity into use and providing professional staffing, this model provides high-intensity, 7-h separate care, guaranteeing parents the Right to Rest.

**Results and discussion:**

Preliminary administrative data (*N* = 45) and in-depth user interviews indicate that this approach has the potential to support parental resilience, potentially functioning as a critical gatekeeper for isolated families. We argue that the strategic repurposing of existing welfare infrastructure offers a cost-effective, scalable framework aimed at mitigating child abuse risks in resource-constrained contexts.

## 1 Introduction

Parenting multiple infants (twins, triplets, etc.) is globally reported to impose significantly higher physical, mental, and economic burdens than parenting a single child (1). Previous studies have shown that the incidence of child abuse in multiple-birth families is several times greater than it is in single-birth families. In Japan, the risk of fatal child abuse among infants from multiple births (excluding murder–suicide cases) has been reported to be 2.5 to 4 times higher than that among single-birth infants, highlighting the severity of this issue and its importance as a public health concern (2, 3). The core of this risk lies in the parents’ chronic sleep deprivation, caused by their care needs and the simultaneous social isolation resulting from the difficulty of going out while ensuring care for the children. Many community child-rearing support measures are designed using a single child model and do not sufficiently cover the extreme daily life challenges of multiple-birth families, for whom even basic physiological needs, such as sleep, bathing, and eating, are hindered (4). The question of how to fill this support gap forms an urgent issue in modern family policy.

**Figure 1:**
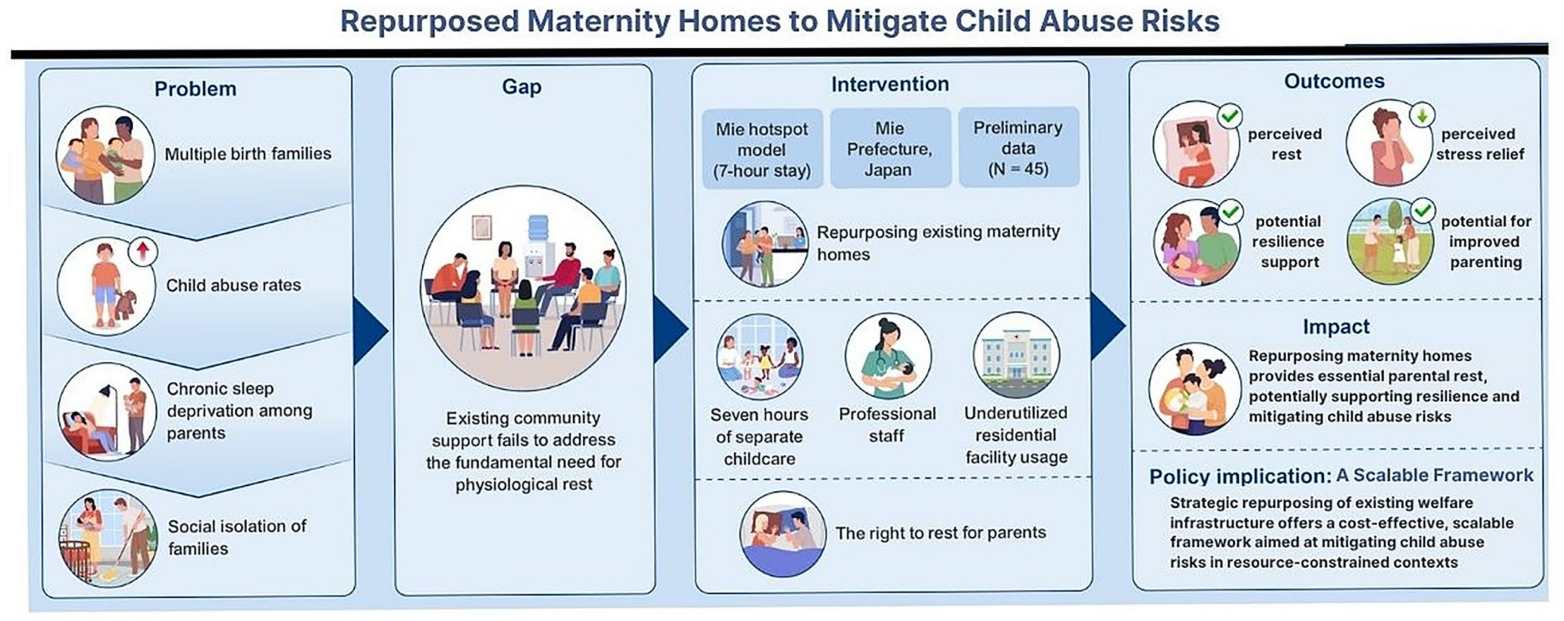
Conceptual framework of the Hot Spot intervention model for supporting multiple-birth families and potentially mitigating child abuse risks.

An innovative model has emerged in Japan’s Mie Prefecture to address this issue. Through this policy, the local government of Mie Prefecture positions high-intensity intervention for multiple-birth families through a strategic reinterpretation of existing social resources. This is the Mie Hot Spot Model, which utilizes maternity homes (residential facilities for mothers and children) as community child-rearing support hubs. While Japanese maternity homes have historically focused on protecting the economically disadvantaged and victims of domestic violence (DV), in recent years, they have undergone multi-functionalization to provide their high level of expertise and residential functions to the community. The Mie Hot Spot Model project optimizes the characteristics of the existing infrastructure, such as 24-h professional staffing and private room environments for the needs multiple-birth families have for rest (Figure 1).

This paper analyzes practical examples in Mie Prefecture and clarifies how the strategic repurposing of existing welfare facilities can potentially mitigate abuse risks in multiple-birth families. In particular, with respect to fulfilling physiological needs that cannot be met in short-term home-visit support, it explores how long-term stay support (7-h day care) can contribute to perceived parental psychological resilience. This study provides important suggestions as a sustainable support model using existing stock for welfare policies in other countries that are facing resource constraints.

The origin of maternity homes dates back to the maternity hostels developed in accordance with the Child Welfare Act, enacted in 1947, soon after the end of the Second World War. Initially, these hostels primarily provided a poverty-relief function for housing security for the benefit of destitute mothers and children, such as war widows and returnees. However, during the period of high economic growth, the social environment around fatherless families underwent a transformation, including decreased levels of bereavement and increased numbers of admissions due to separation or being unmarried.

In the 1990s, professional support came to be required for complex life issues going beyond mere economic deprivation, such as shelter functions for the victims of DV and responding to child-rearing anxiety and child abuse. In response to this, they came to be called maternity homes (*boshi seikatsu shien shisetsu*) in the 1997 revision of the Child Welfare Act. This change was not limited to a change in nomenclature but also a paradigm shift from a protection-centered paradigm to one that placed the independence support of mothers and children at its core.

In the 2004 revision of the Child Welfare Act and in subsequent reviews of social care, maternity homes were called upon to strengthen their community support functions. Muto (5) verified these guidelines and future visions, redefining community support by facilities as an interface to realize seamless support between admission support and community support rather than mere “functional opening of facilities.”

Muto considers that the support provided by facilities is to be understood within a three-layer structure: aftercare (individual support for those who have left the facility), community support (for all child-rearing households in the community), and community collaboration (incorporating community development in cooperation with community members and social resources). In particular, a facility’s effectiveness in providing community social work, including outreach for potential needs in the community and integrally develops professional individual support and community development as the key to the significance of the existence of the facility.

However, the axis of discussion in the review by Muto and others continues to center on approaches to fatherless families and economically disadvantaged households. To the best of our knowledge, no practical research has been conducted that clearly positions multiple-birth families—a group that does not necessarily entail economic deprivation but faces extremely high child-rearing risks—as a strategic target for community social work.

Uzuhashi’s (6) perspective on social exclusion and deprivation of opportunity theoretically supports this practice. Uzuhashi considers that the essence of the experience of households that face poverty and difficulties lies in their being cut off from social relations, functioning as a state of deprivation from certain opportunities (communication, privacy, having time to oneself, etc.) that they would naturally enjoy. Such deprivation is a factor lowering the subject’s self-esteem and wearing down resilience, the power to overcome adversity.

However, a review by Muto and Uzuhashi, shows that the focus of discussion continues to be on fatherless families and economically disadvantaged households. While multiple-birth families are not necessarily directly linked to economic deprivation, they are structurally deprived of opportunities for rest and social participation resulting from their extreme child-rearing environment, a situation that is extremely similar to the structure of social exclusion, as noted by Uzuhashi. Therefore, utilizing maternity homes as resources (interfaces) for multiple-birth support and guaranteeing the basic opportunity for rest can be positioned as a concrete form of a practice of social inclusion to structurally support the recovery of parents’ resilience.

## 2 Methods

### 2.1 Case overview

#### 2.1.1 Target case: Mie prefecture maternal and newborn Hot Spot Model project

The Mie Hot Spot Model project falls under the jurisdiction of the Department of Child and Welfare of Mie Prefecture and is being implemented through the commissioning of two maternity homes in the prefecture (Minori-en in Tsu City and Nanohana-en in Yokkaichi City). The target population for this project is pregnant and postpartum women who are living in the prefecture (for up to approximately 2 years postpartum), but it is noteworthy that it explicitly takes “multiple-birth mothers and others recognized as needing special support” as priority targets.

The service provides day use from 9:00 a.m. to 4:00 p.m., without a usage fee; the service includes lunch. Users can utilize private rooms in the facility (living spaces such as 2DK) and can receive consultation support, guidance in child-rearing skills, and the provision of rest (respite) in mother–child separation by professionals such as midwives and nursery teachers.

#### 2.1.2 Characteristics of multiple-birth support

The background for this project, which explicitly targets multiple-birth families, includes launching the initiative to supplement the difficulty of the use of postpartum care services with multiple-birth infants or accompanied by older siblings and a lack of acceptance facilities. Furthermore, based on the high sense of child-rearing burden and risk of abuse in multiple-birth parenting, there is also an intention to prevent abuse through early connection with high-risk pregnant women. After FY2025, the number of uses is set to the number of children ×4, ensuring more than double the usage quota for multiple-birth families relative to single-birth families.

### 2.2 Methodology

This study employed a mixed-methods explanatory sequential design (QUANT→qual). First, administrative utilization records and user survey data (*N* = 45) were analyzed quantitatively. Second, to explain and further explore the quantitative findings, semi-structured qualitative interviews were conducted with two continuous users of the service. The detailed design of this explanatory sequential approach, including the timing and procedures for integrating the quantitative and qualitative data, is visually summarized in Table 1. This study was approved by the Yokkaichi University Research Ethics Committee (Approval No. 25-9) (see Supplementary Material 1 for the Artificial Intelligence Prompt History/Record of Interactions).

**Table 1:** Mixed-methods integration matrix (QUANT→qual).

Research phase	Quantitative component (QUANT)	Qualitative component (qual)	Integration procedure and purpose
Data collection	Administrative utilization records and user survey data (*N* = 45) focusing on respite care needs.	Semi-structured interviews with two mothers of twins who were continuous users of the service.	Connecting: Quantitative analysis identified high-frequency users, from whom interview participants were purposively selected for in-depth qualitative exploration.
Data analysis	Descriptive statistical analysis of facility utilization rates and the proportion of users from multiple-birth families (22%).	Thematic analysis of interview transcripts to identify key psychological themes.	Building/Side-by-Side Comparison: Quantitative findings regarding the normalization of sleep deprivation provided the basis for examining qualitative themes related to the potential recovery of parental self-efficacy.
Interpretation	High percentage of “rest” needs (70%) and multi-layered relief from both household and childcare responsibilities.	Insights into trust in professional caregivers, reduced psychological barriers to seeking support, and the affordability of the service.	Merging: Quantitative and qualitative findings were integrated in the Discussion to explain how a free, 7-h institutional childcare model can structurally support parental resilience.

## 3 Results

### 3.1 Utilization records and trends in multiple-birth families (quantitative)

The operating rates at both facilities are shifting at a high level; for example, Nanohana-en recorded 91.7% use in May 2024 and Minori-en recorded 95.8% use in the same month. Furthermore, the rate of multiple-birth families is noteworthy. Approximately 22% of all usage is by multiple-birth families, an extremely high utilization density when the population ratio (approximately 1%) is taken into account. In the FY2025 data of “Nanohana-en,” eight out of nine multiple-birth households that used the facility used it repeatedly, and three of these used it at a high frequency, of ten times or more. This suggests that for multiple-birth families, this project functions as an essential aspect of infrastructure for maintaining daily life rather than a temporary event.

### 3.2 Structure of user needs (survey results)

A survey of users of Nanohana-en indicated that the most commonly desired outcome of service was rest, accounting for 70% of the total. This was followed by midwife consultation (12%) and psychological consultation (10%). Before utilization, many mentioned the lack of physiological needs in Maslow’s hierarchy of needs, noting their needs for time alone, sleep, and a slow bath. In multiple-birth parenting, it is normalized that if one parent sleeps, the other is awake, highlighting the situation where guardians can barely perform even basic lifestyle behaviors.

### 3.3 Transformation process by stay-over support (survey results)

Impressions after utilization show that this project offers perceived benefits beyond mere day care. Reports such as “I was able to sleep properly for the first time in a long time,” “I was able to take a bath alone for the first time in several years,” and “I was able to eat a warm meal alone” show that complete rest (complete mother–child separation) can only be obtained by entrusting children to professionals in a safe environment such as this type of facility. Notably, the outcome that “I was released not only from child-rearing but also from housework” shows that the facility environment, where lunch is provided and cleaning is unnecessary, reduces the burden on guardians in multiple ways. In multiple-birth families in particular, ripple effects on child-rearing life following the return home are also reported, including the report that “it makes me feel relaxed after going home” due to receiving bathing assistance at the facility.

### 3.4 Qualitative study results: psychological and physiological transformation process through stay-over support

Analysis of interviews with two continuous users Subject A and Subject B extracted the following three categories as the essential value of this project.

#### 3.4.1 Restoring self from caregiver (time to return to oneself)

Users adopted the 7-h stay not only as mere rest but also as a time to immerse themselves in personal activities or work that had been interrupted by child-rearing. Subject A stated, “I was able to concentrate and push through with creating materials here, which would be interrupted at home,” and Subject B said, “Because people look after them properly, I felt like I returned to myself.” This suggests that this forms a process of supporting the reconstruction of self-efficacy and identity that had been deprived beyond mere day care.

#### 3.4.2 Professional guarantee of peace of mind and elimination of psychological barriers

Trust in professionals was described as a decisive difference from general temporary care or volunteers’ support. Compared to Subject B’s experience of child-rearing policies in non-professional support, where it was stressful, Subject B described the facility staff as “child-rearing pros” and stated that she “could entrust them with peace of mind.” Moreover, while there is resistance to asking relatives to help them by providing time alone the low psychological barrier of being able to ask others casually to have my own time in this project was a factor supporting continuous use.

#### 3.4.3 Importance of physical and economic accessibility

Being able to come empty-handed (diapers and meals being provided) and being free were extremely strong motivations for support among multiple-birth families. Subject B pointed out that, while she shared the household budget with her husband, the use of paid services creates an additional hurdle of consultation with the husband, adding that, because these services were free, she “could step into using it by my own judgment.” This indicates that economic support ensures the autonomy of parents in decision-making.

## 4 Discussion

### 4.1 Guaranteeing the right to rest: maintaining child-rearing functions and mitigating abuse risks

Multiple-birth families are placed into a situation that goes beyond the category of mere child-rearing fatigue and forms a structural crisis where the basic human rights to survive and to rest are threatened.

For community welfare, this crisis is not a problem of individual capacity or ability but is instead a systemic defect, caused by the current child-rearing support system, which is designed with single births as the standard model. To ameliorate extreme care work in multiple-birth parenting, such as where infants cry simultaneously, conventional home-visit support or short-term temporary care does not provide enough resources to guarantee parents’ rest. The provision of 7-h complete mother–child separation and a guarantee of life in a private room provided by this project function as social respite that fills this systemic defect. Physical distancing from the closed child-rearing environment of the home and securing rest in a safe third place forms an indispensable process through which parents can regain human dignity and leeway. As Ochi (7) noted, in multiple-birth parenting support, more than empathetic counseling was needed; instrumental support accompanied by physical burden reduction formed a concrete preventive measure to release parents from being cornered, potentially mitigating the risk of impulsive abuse. While this model uses fulfillment of physiological needs as an entry point, therefore, its reality embodies the strengthening of the safety net aimed at mitigating the risk of family collapse.

### 4.2 Distinction from postpartum care services: filling the policy gap

It is critical to distinguish this model from the standard postpartum care services enshrined in law in 2021. While both provide support to mothers, there are key structural differences. First, general postpartum care tends to emphasize a didactic/healthcare approach, providing breastfeeding management and skills acquisition. However, as Ochi (7) notes, for parents who have no memory of sleeping, educational guidance is ineffective until sleep deprivation is resolved. Unlike the medical model, the Mie Hot Spot Model prioritizes instrumental support (physical care) over education in addressing this urgent need. Second, with respect to staffing, medical institutions often face systemic barriers in accepting multiple-birth infants due to their rigid staffing ratios. In contrast, maternity homes can flexibly arrange for high-density staffing (e.g., 1:2 or 1:3 ratios) with their existing workforce, providing a strength not found in standard care. Third, most postpartum care ends at 1 year. This model extends it to 2 years, covering the high-risk period when infants’ mobility increases, thereby filling the medium-term gap that medical support often misses.

### 4.3 Improving help-seeking capability and preventing isolation through trust-building with professionals

Another important function of this project is found in the improvement of the parents’ help-seeking capability (*juen-ryoku*) through continuous involvement with professionals (nursery teachers, mother and child support workers, etc.) of the maternity home.

Multiple-birth families tend to lose touch with their community and can easily fall into social isolation due to the difficulty of going out and hesitation toward entering their surroundings. In an isolated environment, parents bottle up their child-rearing worries, risking falling into a vicious cycle of losing even the energy to seek support. However, through stay-over support such as that provided by this project, parents have the opportunity to communicate naturally with professionals before and after they take a rest or during their stay.

The reports provided in the interview survey, including that “the staff saw me off warmly” and “I could feel at ease by leaving it to the pros,” show that the use of the facility is not just a day care service, becoming a secure base for parents. This trust relationship (rapport) with professionals is an important aspect of social capital for parents to regain the sense that they are not alone and can appropriately send out SOS signals when they face difficulties. Therefore, this project can be said to function as a gatekeeper, reconnecting closed homes to the community through having professionals become companions for the parents, not just providing rest.

### 4.4 Overcoming systemic exclusion and supporting social inclusion

The prevention of isolation noted in the previous section is reinforced on the systemic side. Behind the hesitation of multiple-birth families to use community child-rearing support centers lies an invisible exclusion, such as movement restrictions with twin strollers and in the eyes of neighbors. This project brought these families back to the framework of social inclusion, redefining existing maternity homes as places where multiple-birth infants can also enter. The fact that utilization is possible from the prenatal period (pregnant women) makes it extremely effective as a preventive intervention. High-risk, multiple-birth pregnant women connecting with the place and professionals of the facility from before giving birth bring them a safety net to prevent postpartum isolation.

In the interview, Subject B stated, “I was not good at just child-rearing and wanted to be connected with society,” suggesting that the use of this project was a turning point for maintaining contact with society. In addition, the physical possibility of being able to go empty-handed without holding diapers or milk and the psychological warmth expressed by all the staff seeing me off when going home was confirmed to alleviate the difficulty of going out that is unique to multiple-birth families. This is evidence that the facility fulfills the function of facilitating the inclusion of families that tend to be isolated into the community by removing physical and psychological barriers to this inclusion.

### 4.5 Governance’s optimal solution: strategic repurposing of existing stock

The policy originality of this model lies in its matching of the surplus resources of maternity homes, which faced a decline in utilization rates, with the modern niche and serious needs of multiple-birth support without requiring the construction of new facilities. This provides a form of extremely sustainable governance enabling rapid policy deployment through repurposing existing resources in local governments with severe resource constraints.

### 4.6 Research limitations

Several limitations should be considered in interpreting the results of this study. First, the study subjects were limited to two mothers who had used this project five or more times. While purposive sampling was used to capture the deep, perceived transformations enabled by the support, the difficulties and needs of those who interrupted use or never reached utilization fell outside the scope of this study. Future research should expand the sample size to incorporate multiple-birth families with various backgrounds.

Second, the study was conducted within a single region, Mie Prefecture, where specific maternity homes are proactively opening their services to the community. Because the installation status of maternity homes and community welfare infrastructure differ across local governments, additional verification in other regions or city scales is required to ensure the universal applicability (generalizability) of this model.

Third, and most importantly, this study is inherently exploratory. Given the limited qualitative sample and the absence of validated psychological outcome measures, comparison groups (control groups), or longitudinal follow-up, this evaluation primarily captures the feasibility, acceptability, and perceived benefits of the Mie Hot Spot Model. The current data do not directly demonstrate definitive effects on long-term child abuse prevention, parental resilience, or sustainable social inclusion. Future work must utilize a longitudinal research design and quantitative outcome tracking to rigorously evaluate the objective effectiveness of this project in reducing fatal abuse and inappropriate parenting over time.

## 5 Conclusion

In this paper, taking the Hot Spot Model in Mie Prefecture as an example, we showed that the multifunctionalization of maternity homes is a highly promising approach for supporting multiple-birth families. In conclusion, the following three points can be highlighted.

First, the life-support functions (bath, meal, private room) and the professional functions of maternity homes have a high affinity with the serious respite needs of multiple-birth families. Second, support for long-term stay with no economic burden (free) fulfills the physiological needs of users and is perceived to contribute to the recovery of child-rearing motivation and the potential mitigation of abuse risks. Third, this model, which used existing infrastructure, can be deployed as an immediate social policy in local governments with resource constraints.

The verification of the financial sustainability of this project and follow-up surveys regarding users’ long-term outcomes (children’s development and transitions in parents’ mental health) are needed in future research. As described above, this study clarified the reality of the multi-layered difficulties that are faced by multiple-birth parenting from the field of community welfare, which is not found in conventional multiple-birth parenting research, which leads to the proposal of a necessary support model that is more universal, reaching a wider area. There is no doubt that the vector from facility to community that is shown by the case of Mie Prefecture and the inclusion of the target from single-birth to multiple-birth will be an important model case for future family policy.

## Data Availability

The original contributions presented in the study are included in the article/supplementary material, further inquiries can be directed to the corresponding author.
